# The effect of tonsillectomy with adenoidectomy on medical services used in association with otitis media based on Korean national sample cohort data

**DOI:** 10.1186/s12962-020-00243-7

**Published:** 2020-10-27

**Authors:** Junhui Jeong, Jung Kyu Choi, Jae Sung Nam, Hyang Ae Shin, Jung Hyun Chang, Hyun Seung Choi

**Affiliations:** 1 Department of Otorhinolaryngology, National Health Insurance Service Ilsan Hospital, 100 Ilsan-ro, Ilsandong-gu, Goyang, 10444 Korea; 2 Department of Policy Research Affairs, National Health Insurance Service Ilsan Hospital, Goyang, Korea; 3 Department of Otorhinolaryngology, Yonsei University College of Medicine, Seoul, Korea

**Keywords:** Tonsillectomy, Adenoidectomy, Otitis media, Medical service, Population-based study

## Abstract

**Background:**

The effect of tonsillectomy with adenoidectomy (T&A) on otitis media has been investigated, but there have been no reports of the relationship between T&A and medical services used in association with otitis media. We investigated the effect of T&A on otitis media with regard to the number and cost of medical services used.

**Methods:**

From the National Health Insurance Service National Sample Cohort data in Korea, we selected patients 7 years old or younger in 2002 who had T&A in 2005 while between the ages of three and ten. A control group was established matching the patient group with similar propensities of demographic characteristics. The number and cost of medical services used in association with otitis media were analyzed for 3 years before T&A through 8 years after T&A.

**Results:**

The total number of patients was 1,338, with 227 in the T&A group and 1,111 in the non-T&A group. The number of medical services used was not significantly different between the T&A and non-T&A groups before and after surgery. The cost of medical services used was significantly higher in the T&A group than in the non-T&A group one year before surgery. The cost of medical services used was not significantly different between the two groups after surgery.

**Conclusions:**

There were no significant differences between the T&A and non-T&A groups in the number and cost of medical services used in association with otitis media after surgery.

## Background

Tonsillectomy with adenoidectomy (T&A) is a commonly performed surgery for children in otorhinolaryngologic departments [1–5]. Frequent tonsillitis and obstructive sleep apnea are indications for T&A [3–5].

Otitis media is also a common disease in children [6]. Studies that have explored the effect of tonsillectomy or adenoidectomy on otitis media have specifically investigated disease occurrence, the disease-free period, and the rate of ventilation tube insertion [1, 3, 7–10]. Although several studies have reported reductions in health care costs or visits in association with obstructive sleep apnea syndrome or in general after T&A [11, 12], there have been no reports of the relationship between tonsillectomy or adenoidectomy and medical services used in association with otitis media.

We used data from the Korean National Health Insurance Service National Sample Cohort to investigate the relationship between T&A and medical services used in association with otitis media. This study focused on the number and cost of medical services used. Although the effect of T&A on otitis media has been demonstrated in terms of the disease itself, here we concentrate on the effect of T&A from the perspective of cost-effectiveness.

## Methods

### Subjects

This study includes data from the National Health Insurance Service National Sample Cohort in Korea, which is a two-percent representative subsample from the entire National Health Insurance Service database. From the national sample cohort data, we selected patients who were 7 years old or younger in 2002, had T&A in 2005 while between the ages of three and ten, and were tracked from 2002 to 2013. We excluded patients who had T&A in any year other than 2005. All people in Korea are obligated to join the National Health Insurance Service. Accordingly, the approximately one million total subjects in the national sample cohort each year represent about two percent of the Korean population (50 million people). Therefore, the results in this study present a fair representation of the entire Korean population. The control group was composed of people who did not have T&A between 2002 and 2013, and was selected to match the T&A group demographics for sex, age, income level, residence, type of National Health Insurance, and medical services used in association with otitis media during the 3 years before T&A in 2005. The two groups had similar demographic characteristics. Individual characteristics that can influence the incidence of otitis media (e.g., past medical and surgical history, family history, allergies, the presence of comorbidities) and surgeries other than T&A (e.g., tympanostomy tube insertion) were not considered, but their effects may have been negligible given the use of a propensity-matched control group and the large sample size.

### Design

**Fig. 1:**
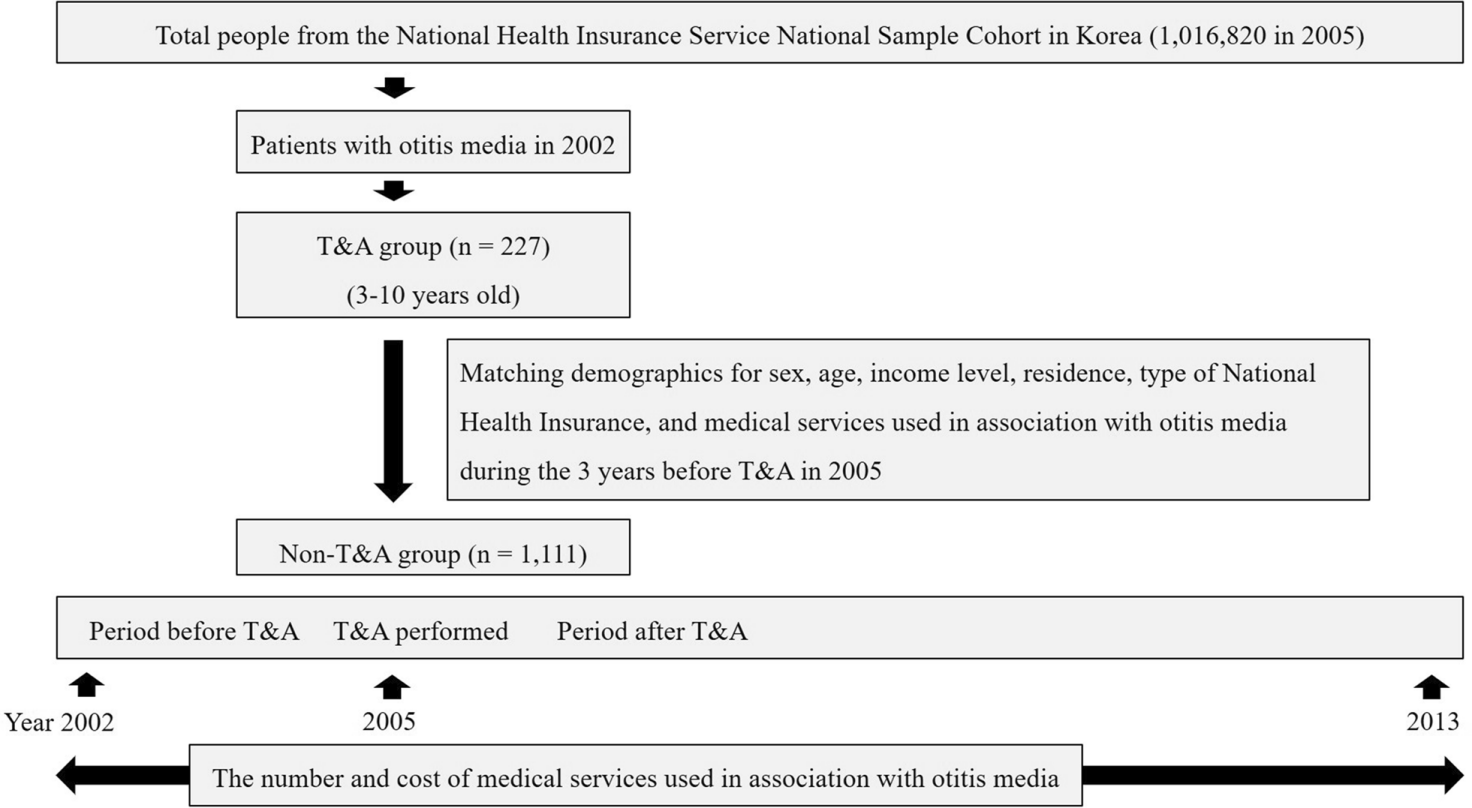
Flow of the study. T&A, tonsillectomy with adenoidectomy

From 3 years before T&A through 8 years after T&A, the number and cost of medical services used in association with otitis media, which included acute otitis media and otitis media with effusion, were analyzed. The analysis used the main diagnostic codes from the International Classification of Diseases (ICD) that were recorded during each patient’s clinic visits. The number of medical services used in association with otitis media means the number of visits in which the main diagnostic codes were associated with otitis media. The cost of medical services used in association with otitis media is defined as the cost of all visits in which the main diagnostic codes were associated with otitis media. Patient age at the time of T&A was divided into four groups (3–4 years old, 5–6 years old, 7–8 years old, and 9–10 years old), and the number and cost of medical services used were compared among these age groups. Costs were not adjusted for inflation. The flow of the study is depicted in Fig. 1.

### Definition of the disease

The main diagnostic codes associated with otitis media that are found in the ICD were (1) H65 (nonsuppurative otitis media), (2) H66 (suppurative and unspecified otitis media), and (3) H67 (other otitis media).

### Statistical analysis

Chi square tests were used to compare demographic characteristics of patients with otitis media in 2002 between the T&A group and the non-T&A group. The number and cost of medical services used in association with otitis media in each period was compared between groups with t tests. Statistical analyses were performed with SAS 9.3 (SAS Inc., Cary, NC, USA). *P* < 0.05 was considered significant.

## Results

### Otitis media status in 2002 according to T&A status in 2005

Demographic characteristics of patients with otitis media in 2002 according to T&A status in 2005 were analyzed (Table 1). The total number of patients was 1,338, with 227 in the T&A group and 1111 in the non-T&A group. There were more male patients (62.8%) than female patients (37.2%), and more than half of the patients were 2–5 years old (72.6%). There were no significant differences between the two groups in sex, age, income, type of National Health Insurance, or residence.

**Table 1:** Demographic characteristics of patients with otitis media in 2002

	Number (%)	Non-T&A group	T&A group	*P* value
Total patients with otitis media	1338 (100)	1111	227	
Sex				
Male	840 (62.8)	700	140	0.7051
Female	498 (37.2)	411	87	
Age in 2002				
0–1 year old	85 (6.4)	70	15	0.9936
2–3 years old	547 (40.9)	455	92	
4–5 years old	424 (31.7)	353	71	
6–7 years old	282 (21.1)	233	49	
Income level				
1st (lowermost) quintile	92 (6.9)	80	12	0.7896
2nd quintile	139 (10.4)	112	27	
3rd quintile	326 (24.4)	272	54	
4th quintile	413 (30.9)	341	72	
5th (uppermost) quintile	368 (27.5)	306	62	
Type of National Health Insurance				
Employee member	813 (60.8)	674	139	0.8732
Local member	525 (39.2)	437	88	
Residence				
Seoul (capital)	222 (16.6)	179	43	0.7429
Metropolitan	338 (25.3)	281	57	
City (small and medium sized)	711 (53.1)	594	117	
County	67 (5.0)	57	10	

*T&A* Tonsillectomy with adenoidectomy

### Differences in the number of medical services used in association with otitis media according to T&A status

The number of medical services used in association with otitis media was analyzed according to T&A status in 2005. Data were analyzed from 3 years before T&A until 8 years after surgery. The number of medical services used in association with otitis media was not significantly different between the T&A and non-T&A groups before and after surgery. The number was highest 1 year before surgery. As the age of patients receiving T&A increased, the number of medical services used in association with otitis media decreased (Table 2).

**Table 2:** The number of medical services used in association with otitis media

Age in 2005	Period before or after T&A	3 years before	2 years before	1 year before	1 year after	2 years after	3 years after	4 years after	5 years after	6 years after	7 years after	8 years after
Total	Non-T&A (B)	0.86	1.16	1.23	0.70	0.47	0.66	0.51	0.35	0.26	0.24	0.22
	T&A (A)	1.02	1.16	1.26	0.81	0.52	0.89	0.79	0.40	0.37	0.11	0.15
	Difference(A-B)	0.16	0.00	0.03	0.11	0.05	0.24	0.28	0.06	0.12	−0.13	−0.07
	*P* value	0.269	0.992	0.863	0.445	0.630	0.258	0.198	0.631	0.300	*0.010*	0.331
3-4 years old	Non-T&A (B)	1.03	1.74	2.77	1.54	1.09	2.36	0.89	0.54	0.31	0.04	0.21
	T&A (A)	1.27	1.87	2.53	1.80	0.80	1.00	1.00	0.67	0.47	0.07	0.53
	Difference(A-B)	0.24	0.12	−0.24	0.26	−0.29	−1.36	0.11	0.12	0.15	0.02	0.32
	*P* value	0.758	0.875	0.803	0.767	0.420	0.157	0.899	0.861	0.632	0.697	0.299
5-6 years old	Non-T&A (B)	1.03	1.54	1.57	1.05	0.65	0.81	0.67	0.40	0.37	0.30	0.24
	T&A (A)	1.26	1.49	1.58	1.16	0.71	1.18	1.08	0.38	0.53	0.17	0.06
	Difference (A-B)	0.23	−0.05	0.01	0.11	0.06	0.37	0.40	−0.02	0.16	−0.13	−0.19
	*P* value	0.379	0.885	0.973	0.728	0.774	0.341	0.397	0.894	0.468	0.182	*0.002*
7-8 years old	Non-T&A (B)	0.89	1.02	0.99	0.36	0.25	0.37	0.36	0.29	0.18	0.27	0.23
	T&A (A)	0.77	1.07	0.83	0.41	0.36	0.96	0.87	0.52	0.30	0.07	0.23
	Difference(A-B)	−0.12	0.05	−0.17	0.05	0.11	0.58	0.51	0.23	0.13	−0.20	0.01
	*P* value	0.530	0.867	0.550	0.703	0.560	0.116	0.080	0.288	0.406	*0.035*	0.975
9-10 years old	Non-T&A (B)	0.43	0.48	0.46	0.30	0.25	0.27	0.30	0.27	0.13	0.14	0.18
	T&A (A)	0.86	0.47	0.90	0.45	0.31	0.24	0.08	0.20	0.14	0.06	0.08
	Difference(A-B)	0.43	−0.01	0.43	0.15	0.06	−0.03	−0.21	−0.07	0.01	−0.08	−0.09
	*P* value	0.122	0.972	0.253	0.387	0.719	0.890	0.069	0.731	0.880	0.200	0.405

*T&A* tonsillectomy with adenoidectomy

Italic indicates *P* value < 0.05

### Differences in the cost of medical services used in association with otitis media according to T&A status

The cost of medical services used in association with otitis media was analyzed according to T&A status in 2005. Data were analyzed from 3 years before T&A until 8 years after surgery. The cost of medical services used in association with otitis media was significantly higher in the T&A group [128,041 Korean Won (KRW), or about 128 United States Dollars (USD)] than in the non-T&A group (31,307 KRW, or about 31 USD) in 1 year before surgery. After surgery, there were no significant differences between the two groups (Table 3).

**Table 3:** The cost of medical services used in association with otitis media

Age in 2005	Period before or after T&A	3 years before	2 years before	1 year before	1 year after	2 years after	3 years after	4 years after	5 years after	6 years after	7 years after	8 years after
Total	Non-T&A (B)	25,627	30,854	31,307	19,943	15,802	8660	8613	4450	3221	3478	5712
	T&A (A)	27,231	32,552	128,041	29,397	12,812	10,353	11,248	5232	4716	3269	2561
	Differences (A-B)	1604	1698	96,735	9454	v2990	1692	2635	783	1496	−209	−3151
	*P* value	0.721	0.750	*<0.001*	0.089	0.524	0.493	0.561	0.603	0.261	0.913	0.167
3-4 years old	Non-T&A (B)	38,446	56,065	76,974	56,097	53,905	26,082	9159	7042	3785	566	2517
	T&A (A)	31,353	44,321	301,891	63,005	16,618	11,605	10,499	7495	8463	1043	11,423
	Differences (A-B)	−7093	−11,744	224,917	6908	−37,287	−14,477	1340	453	4678	476	8907
	*P* value	0.742	0.533	*0.010*	0.802	0.239	0.179	0.882	0.945	0.511	0.668	0.299
5-6 years old	Non-T&A (B)	30,588	43,603	38,732	26,306	17,152	10,583	14,552	5450	4674	3815	3569
	T&A (A)	35,905	37,629	151,749	41,379	19,278	12,864	16,506	4227	5900	6255	952
	Differences (A-B)	5316	−5974	113,017	15,073	2126	2281	1954	−1224	1227	2440	−2616
	*P* value	0.545	0.482	*< 0.001*	0.129	0.742	0.624	0.856	0.510	0.618	0.591	*0.004*
7-8 years old	Non-T&A (B)	22,835	22,062	23,036	6814	13,514	4584	4271	3553	2245	3725	8658
	T&A (A)	21,184	39,552	95,650	20,627	9389	12,049	11,332	6378	4110	1519	3376
	Differences (A-B)	−1651	17,490	72,614	13,813	−4125	7465	7060	2825	1865	−2206	−5282
	*P* value	0.804	0.137	*0.010*	0.053	0.692	0.057	*0.040*	0.258	0.332	0.111	0.392
9-10 years old	Non-T&A (B)	16,315	11,704	15,617	16,544	5184	5848	3431	3075	1691	3322	6392
	T&A (A)	18,445	9274	77,243	9319	4467	2796	1485	4768	2225	881	1688
	Differences (A-B)	2130	−2431	61,627	−7225	−718	−3052	−1946	1693	534	−2441	−4705
	*P* value	0.816	0.600	0.056	0.531	0.766	0.383	0.242	0.693	0.680	0.096	0.310

*T&A* tonsillectomy with adenoidectomy

Data are presented as Korean Won (KRW), italic indicates *P* value < 0.05

## Discussion

The efficacy of tonsillectomy, adenoidectomy, and T&A on otitis media has been investigated in many studies. Currently in the United States, tympanostomy tubes, adenoidectomy, or both are recommended for otitis media with effusion in patients age 4 or older based on several systematic reviews [6, 13–15]. Adenoidectomy is not recommended in patients younger than 4 if there are no distinct indications such as nasal obstruction or chronic adenoiditis [6].

The pathophysiology of otitis media includes dysfunction in ventilation, drainage of secretion, and mucosa edema due to negative pressure caused by Eustachian tube dysfunction. Adenoidectomy may enhance Eustachian tube function, which would improve ventilation and drainage and help control pressure in the middle ear. In contrast, tonsillectomy alone may not be a helpful treatment for otitis media because tonsils are not anatomically associated with Eustachian tube function. Biofilms of bacteria in the adenoid have been reported to cause inflammation and mucosal edema, resulting in otitis media [5, 16, 17]. Paradise et al. suggested that otitis media could be associated with infection not only in the nasopharynx, but also in the oropharynx based on their results that T&A was narrowly more efficacious against otitis media than adenoidectomy only [1].

The effect of T&A on the use of medical services has been reported in several studies. Tarasiuk et al. reported reductions in total annual health care costs, number of new admissions, emergency department visits, number of consultations, and prescribed drugs in children with obstructive sleep apnea syndrome who had T&A [11]. Using Medicaid data from the United States, Chang et al. reported a reduction in costs after T&A in children with adenotonsillar hypertrophy due to less antibiotic use and fewer outpatient visits [12]. However, these studies analyzed visits and the costs of diseases other than otitis media. Furthermore, the characteristics of patients included in our study are different from those of previous studies. We included patients from a national database that included a representative population with data on whole-household income.

We did not find any significant differences in number of medical services used in association with otitis media between surgery and non-surgery groups before and after surgery. Similarly, the cost of medical services used in association with otitis media was not significantly different between the two groups after surgery. Medical service costs, however, were significantly higher in the surgery group 1 year before surgery. Overall, T&A did not appear to help decrease the number or cost of medical services used in association with otitis media.

The abrupt increase in the cost of medical services used in association with otitis media observed in the T&A group but not the non-T&A group 1 year before surgery is difficult to explain. It could be attributable to the cost associated with preoperative workups before undergoing general anesthesia, including complete blood count, blood chemistry, chest plain radiography, and electrocardiography. Although these costs are not directly associated with otitis media, many of the patients who underwent preoperative workups might have had accompanying otitis media, and the main diagnostic codes of some patients might have been the codes associated with otitis media. No medical services except preoperative workups could have caused such an abrupt increase in costs just before surgery.

Considering this, the continued decrease in cost in the T&A group immediately after surgery does not demonstrate effectiveness of T&A for otitis media. If T&A were effective for otitis media, both the number and cost of medical services used in association with otitis media would become significantly lower in the T&A group than in the non-T&A group after surgery. The cost of medical services used in association with otitis media decreased with time after surgery in both groups. This could be attributed to a spontaneous decrease of otitis media occurrence. Based on the results of this study, it seems that T&A is not related to otitis media regarding number and cost of medical services.

Although this study was retrospective in nature and based on National Health Insurance Service data, and although the data were analyzed by diagnostic codes, we believe that the relationships between T&A and otitis media with respect to medical services used were effectively evaluated for a sample cohort that represented the wider population. The T&A and non-T&A groups in this study had similar sociodemographic characteristics, and the object of the study was to investigate differences in the number and cost of medical services used by the two groups. Thus, the effects of other factors, such as sex, age, income, residence, and factors that can influence the incidence of otitis media (such as allergies and other comorbidities), were not investigated using multivariate regression analysis. Also, detailed costs for consultation fees, workups, and treatment procedures could not be compared between the two groups, as the National Sample Cohort database only contained data on the total cost of medical services used in association with otitis media. Nonetheless, this research is significant because it is the first study to use population-based data to investigate the effect of T&A on the use of medical services related to otitis media.

Patients who underwent adenoidectomy alone or tonsillectomy alone were not included in this study because there were few of them. A future study that designates more surgery classifications (e.g., tonsillectomy alone, adenoidectomy alone, and T&A) may provide more meaningful results. Population-based studies may also be important for national health authorities to identify medical services used in association with otitis media and to manage national health insurance funds appropriately.

## Conclusions

There were no significant differences between the T&A and non-T&A groups in the number of medical services used in association with otitis media before and after surgery. There were also no significant differences between the groups in the cost of medical services used in association with otitis media after surgery.

## Data Availability

The datasets used and/or analysed during the current study are available from the corresponding author on reasonable request.

## References

[1] Paradise JL, Bluestone CD, Colborn DK (1999). Adenoidectomy and adenotonsillectomy for recurrent acute otitis media: parallel randomized clinical trials in children not previously treated with tympanostomy tubes. JAMA.

[2] Van Den Akker EH, Hoes AW, Burton MJ, Schilder AG (2004). Large international differences in (adeno)tonsillectomy rates. Clin Otolaryngol Allied Sci.

[3] Oomen KP, Rovers MM, van den Akker EH, van Staaij BK, Hoes AW, Schilder AG (2005). Effect of adenotonsillectomy on middle ear status in children. Laryngoscope.

[4] Gigante J (2005). Tonsillectomy and adenoidectomy. Pediatr Rev.

[5] Ramos SD, Mukerji S, Pine HS (2013). Tonsillectomy and adenoidectomy. Pediatr Clin North Am.

[6] Rosenfeld RM, Shin JJ, Schwartz SR (2016). Clinical Practice Guideline: otitis Media with Effusion (Update). Otolaryngol Head Neck Surg.

[7] Maw AR (1983). Chronic otitis media with effusion (glue ear) and adenotonsillectomy: prospective randomised controlled study. Br Med J.

[8] Maw R, Bawden R (1993). Spontaneous resolution of severe chronic glue ear in children and the effect of adenoidectomy, tonsillectomy, and insertion of ventilation tubes (grommets). BMJ.

[9] Paradise JL, Bluestone CD, Rogers KD, et al. Efficacy of adenoidectomy for recurrent otitis media in children previously treated with tympanostomy-tube placement. results of parallel randomized and nonrandomized trials. JAMA. 1990. 263(15):2066-73. 2181158

[10] Kadhim AL, Spilsbury K, Semmens JB, Coates HL, Lannigan FJ (2007). Adenoidectomy for middle ear effusion: a study of 50,000 children over 24 years. Laryngoscope.

[11] Tarasiuk A, Simon T, Tal A, Reuveni H (2004). Adenotonsillectomy in children with obstructive sleep apnea syndrome reduces health care utilization. Pediatrics.

[12] Chang JJ, Buchanan P, Geremakis C, Sheikh K, Mitchell RB (2014). Cost analysis of tonsillectomy in children using medicaid data. J Pediatr.

[13] Boonacker CW, Rovers MM, Browning GG, Hoes AW, Schilder AG, Burton MJ (2014). Adenoidectomy with or without grommets for children with otitis media: an individual patient data meta-analysis. Health Technol Assess.

[14] Mikals SJ, Brigger MT (2014). Adenoidectomy as an adjuvant to primary tympanostomy tube placement: a systematic review and meta-analysis. JAMA Otolaryngol Head Neck Surg.

[15] Wallace IF, Berkman ND, Lohr KN, Harrison MF, Kimple AJ, Steiner MJ (2014). Surgical treatments for otitis media with effusion: a systematic review. Pediatrics.

[16] Andreoli SM, Schlosser RJ, Wang LF, Mulligan RM, Discolo CM, White DR (2013). Adenoid ciliostimulation in children with chronic otitis media. Otolaryngol Head Neck Surg.

[17] Saafan ME, Ibrahim WS, Tomoum MO (2013). Role of adenoid biofilm in chronic otitis media with effusion in children. Eur Arch Otorhinolaryngol.

